# Implications of O-glycan modifications in the hinge region of a plant-produced SARS-CoV-2-IgA antibody on functionality

**DOI:** 10.3389/fbioe.2024.1329018

**Published:** 2024-03-06

**Authors:** Pia Uetz, Kathrin Göritzer, Emil Vergara, Stanislav Melnik, Clemens Grünwald-Gruber, Rudolf Figl, Ala-Eddine Deghmane, Elisabetta Groppelli, Rajko Reljic, Julian K.-C. Ma, Eva Stöger, Richard Strasser

**Affiliations:** ^1^ Department of Applied Genetics and Cell Biology, Institute of Plant Biotechnology and Cell Biology, University of Natural Resources and Life Sciences, Vienna, Austria; ^2^ Institute for Infection and Immunity, St George’s University of London, London, United Kingdom; ^3^ Core Facility Mass Spectrometry, University of Natural Resources and Life Sciences, Vienna, Austria; ^4^ Invasive Bacterial Infections Unit, Institut Pasteur, Université Paris Cité, Paris, France

**Keywords:** antibody, glycoprotein, glycosylation, *Nicotiana benthamiana*, posttranslational modification, virus

## Abstract

**Introduction:** Prolyl-4-hydroxylases (*P4H*) catalyse the irreversible conversion of proline to hydroxyproline, constituting a common posttranslational modification of proteins found in humans, plants, and microbes. Hydroxyproline residues can be further modified in plants to yield glycoproteins containing characteristic O-glycans. It is currently unknown how these plant endogenous modifications impact protein functionality and they cause considerable concerns for the recombinant production of therapeutic proteins in plants. In this study, we carried out host engineering to generate a therapeutic glycoprotein largely devoid of plant-endogenous O-glycans for functional characterization.

**Methods:** Genome editing was used to inactivate two genes coding for enzymes of the *P4H10* subfamily in the widely used expression host *Nicotiana benthamiana*. Using glycoengineering in plants and expression in human HEK293 cells we generated four variants of a potent, SARS-CoV-2 neutralizing antibody, COVA2-15 IgA1. The variants that differed in the number of modified proline residues and O-glycan compositions of their hinge region were assessed regarding their physicochemical properties and functionality.

**Results:** We found that plant endogenous O-glycan formation was strongly reduced on IgA1 when transiently expressed in the *P4H10* double mutant *N. benthamiana* plant line. The IgA1 glycoforms displayed differences in proteolytic stability and minor differences in receptor binding thus highlighting the importance of O-glycosylation in the hinge region of human IgA1.

**Discussion:** This work reports the successful protein O-glycan engineering of an important plant host for recombinant protein expression. While the complete removal of endogenous hydroxyproline residues from the hinge region of plant-produced IgA1 is yet to be achieved, our engineered line is suitable for structure-function studies of O-glycosylated recombinant glycoproteins produced in plants.

## 1 Introduction

Since the advent of biotechnology and recombinant production of therapeutic proteins in the 1980s, the importance of biologics for the treatment of various diseases ranging from infectious diseases to cancer and metabolic disorders is ever-increasing, making them one of the fastest-growing segments of the pharmaceutical industry (Owczarek et al., 2019). More than two-thirds of the over 220 marketed therapeutic proteins and peptides are glycoproteins (Li and d’Anjou, 2009), with much more currently in the development pipeline (Whaley and Zeitlin, 2022). Glycosylation is the most prevalent posttranslational modification (PTM) in natural and biopharmaceutical proteins and was shown to influence production-relevant parameters such as protein folding and functional properties, such as proteolytic stability, bioactivity, and *in vivo* half-life (Arnold et al., 2007; Durocher and Butler, 2009; Strasser, 2023). Oligosaccharides attached to peptides via asparagine (Asn) are referred to as N-glycans, whereas O-glycosylation encompasses sugar residues linked to the hydroxyl group of threonine (Thr), serine (Ser) hydroxylysine or hydroxyproline (HyP) (Strasser et al., 2021).

The functional activity of many therapeutic glycoproteins is dependent on glycosylation; hence they are produced in recombinant expression systems that can perform this PTM. Plants offer high scale-up potential with the ability to carry out most of the PTMs found in mammalian cells. Their endogenous N- and O-glycosylation patterns differ from humans, which has hampered the broad application of the expression system to produce human therapeutics (Strasser et al., 2021). While there has been considerable effort in glycoengineering popular expression hosts such as *Nicotiana benthamiana*, *N. tabacum*, tobacco BY2 cells and the moss *Physcomitrella patens* towards humanized- and customized N-glycosylation (Bakker et al., 2001; Koprivova et al., 2004; Strasser et al., 2008; Kallolimath et al., 2016; Limkul et al., 2016; Mercx et al., 2017; Jansing et al., 2019; Bohlender et al., 2020; Herman et al., 2021; Göritzer et al., 2022; Kogelmann et al., 2023) limited attention was cast towards modulating the plant endogenous O-glycosylation pathway (Castilho et al., 2012; Yang et al., 2012; Parsons et al., 2013; Dicker et al., 2016; Ramírez-Alanis et al., 2018; Mócsai et al., 2021; Uetz et al., 2022). Plant-endogenous HyP and further modifications with pentoses (arabinoses) were found in several recombinantly produced proteins such as IgA1, MUC1, EPO-Fc, and Ara h 2 (Karnoup et al., 2005; Weise et al., 2007; Pinkhasov et al., 2011; Castilho et al., 2012; Yang et al., 2012; Üzülmez et al., 2021). The impact of these plant-specific HyP residues and attached pentoses on the function and potency of therapeutic proteins is currently unknown. These non-human glycans could be immunogenic or contribute to allergic reactions through IgE-binding epitopes (Leonard et al., 2005).

While the extent of N-glycosylation is predictable from the amino acid sequence of a specific protein, there has yet to be a consensus motif identified for O-glycosylation, partly explaining the heterogeneity of this PTM (Bennett et al., 2012). Mucin-type O-glycans constitute the most prevalent O-glycosylation forms on secretory human proteins. The biosynthesis occurs in a stepwise manner and starts with the addition of N-acetylgalactosamine (GalNAc)-residues to hydroxyl groups of Ser or Thr and is dependent on the structural properties of the protein (Steen et al., 1998). The plethora of enzymes involved, from the large family of initiating polypeptide GalNAc-transferases to specific glycosyltransferases for chain elongation and branching steps, make the resulting mucin-type O-glycans highly heterogeneous (Tarp and Clausen, 2008). Plant endogenous O-glycosylation happens mostly on hydroxyl groups of HyP onto which arabinose residues are attached via specific arabinosyltransferases in the Golgi, rendering the resulting O-glycoforms highly different from their mammalian counterparts (Gomord et al., 2010; Strasser et al., 2021). The initial conversion from Pro to HyP is carried out in the endoplasmic reticulum (ER) by prolyl-4-hydroxylases (P4Hs), a vast but little-investigated enzyme family in plants (Taylor et al., 2012). Several P4H members from *Arabidopsis thaliana*, tobacco, and *Solanum lycopersicum* have been identified and characterized (Yuasa et al., 2005; Kalaitzis et al., 2010; Velasquez et al., 2011). This paved the way for the discovery of similar enzymes in plant species utilized in plant molecular farming, such as *N. benthamiana* or *P. patens* (Parsons et al., 2013; Mócsai et al., 2021; Uetz et al., 2022). While CRISPR/Cas9-mediated knockout of the P4H4 subfamily in *N. benthamiana* resulted in a noticeable change of the O-glycosylation pattern in the hinge region of a recombinant IgA1 antibody, the presence of considerable amounts of HyP residues suggested that other P4H enzymes are mainly responsible for proline oxidation in the IgA1 hinge region (Uetz et al., 2022). This finding is in line with the characterization of different members of the P4H family from *N. benthamiana* (Mócsai et al., 2021).

Here, we investigated if the targeted knockout of another *NbP4H* subfamily, *NbP4H10,* which is strongly expressed in *N. benthamiana* leaves and displays activity with the hinge peptide (Mócsai et al., 2021), results in a reduction of plant-specific modifications in recombinant IgA1. We investigated the influence of O-glycoform variations introduced by our mutant line on the functional properties of a recombinantly produced SARS-CoV-2 neutralizing IgA1 antibody.

## 2 Materials and methods

### 2.1 Phylogenetic analysis of *NbP4H10* and design of gRNAs

We used the DNA and protein sequence of published *NbP4H10* homolog *Nbv6.1trP31841* (Mócsai et al., 2021) for BLAST search in the *N. benthamiana* genome assemblies of the Sequencing Consortium (NbSC) database (https://www.nbenth.com/), the Queensland University of Technology database (https://benthgenome.qut.edu.au/) and the Sol Genomics Network (https://solgenomics.net/) and aligned resulting sequences via the MAFFT Algorithm L-INS-I (Katoh and Standley, 2013). Suitable target sequences within the two identified *NbP4H10* homologs (see Supplementary Table S1 for precise annotations) were detected by CCTop software (https://cctop.cos.uni-heidelberg.de:8043/) and selected as described previously (Uetz et al., 2022).

We evaluated four CRISPR/Cas9 binary vectors for stable transformation of *N. benthamiana*: pDV107, pDV108, pDV109, and pDV110. These vectors contained two gRNAs each and were assembled and analysed regarding their respective editing efficiencies as described previously (Uetz et al., 2022). After establishing the transient editing efficiencies via *Agrobacterium tumefaciens*-mediated infiltration of *N. benthamiana*, the gRNA module of pDV107 (containing G3 and G7) was transferred to binary vector pBVM5.2 (devoid of a fluorescent protein) to establish the final vector, pB109. All cloning reagents were ordered from Thermo Fisher Scientific (Austria) and primers (Supplementary Table S2) were synthesized by Sigma-Aldrich (Germany).

### 2.2 Transient assay of binary plant vectors

The binary vectors for assaying gRNA efficiencies (pDV107-110) were transformed into chemically competent *Agrobacterium tumefaciens* strain GV3101-pMP90 (Leibniz Institut DSMZ-Deutsche Sammlung von Mikroorganismen und Zellkulturen GmbH, DSM 12364). *N. benthamiana* wildtype plants were infiltrated with transformed Agrobacteria using a syringe and leaf material was collected 5 days post infiltration as described before (Uetz et al., 2022).

Following the isolation of genomic DNA from infiltrated leaf tissues with the NucleoSpin Plant II kit (Macherey-Nagel, Germany), gene regions of both *NbP4H10* homologs targeted by gRNAs were amplified with exon-specific primers (Supplementary Table S2). The presence of mutations was confirmed by Sanger sequencing (Microsynth, Switzerland). Knockout scores for gRNAs were established using TIDE (http://shinyapps.datacurators.nl/tide/) with the settings previously specified (Uetz et al., 2022).

### 2.3 Plant material and growth conditions

Transgenic T0 *N. benthamiana* plants were generated under sterile conditions from LAB wildtype strain cotyledons transformed with *A*. *tumefaciens* and primary transformants were recovered and maintained as described previously (Uetz et al., 2022). Subsequent generations and wildtype plants for gRNA testing or transient protein expression were generated from seeds germinated in soil and maintained in the greenhouse under the same conditions. For the recombinant production of IgG and monomeric IgA2 variants of the antibody under study, glycoengineered *N. benthamiana* ΔXT/FT plants (Strasser et al., 2008) were cultivated as described above, infiltrated at the age of 6-weeks, and maintained in the greenhouse until harvest.

### 2.4 Generation of stable *N. benthamiana* mutants

Double mutant lines of *NbP4H10* homologs were created by *A. tumefaciens* mediated stable transformation of *N. benthamiana* wild-type seedlings with binary vector pB109. Editing efficiencies in Cas9-positive plants (identified by PCR screening) were determined via PCR with exon-specific primers (Supplementary Table S2). Homozygous knockout transformants were propagated and plants of the T1 generation were screened for the absence of the SpCas9 transgene and presence of mutations (Uetz et al., 2022).

### 2.5 Vector design and cloning

The pEAQ-*HT* plant expression vectors containing the alpha heavy chain and kappa light chains of COVA2-15 (QKQ15273.1, QKQ15189.1) were available from a previous study (Göritzer et al., 2024). For the expression of COVA2-15 IgA1 in HEK293-6E cells the codon-optimized genes of the alpha heavy and kappa light chain were flanked with signal peptides MELGLSWIFLLAILKGVQC and MDMRVPAQLLGLLLLWLSGARC, respectively, and synthesized by GeneArt (Thermo Fisher Scientific, Austria). After PCR amplification of the synthesized DNA via primers “Strings_9F (CTTCCGGCTCGTTTGTCTAGA)/Strings_2R (AAA​AAC​CCT​GGC​GGG​ATC​C)” the resulting fragment was inserted via *Xba*I/*Bam*HI restriction cloning into vector pTT5 (National Research Council of Canada).

For the recombinant production of the receptor binding domain (RBD) of the SARS-CoV-2 spike protein in HEK293-6E cells a vector containing a C-terminal 6xHis-tag was obtained from Mark Dürkop (BOKU, Vienna) (Klausberger et al., 2022), and the purification carried out as described before (Göritzer et al., 2024). The construct for mammalian cell-culture-based production of CD89 with a C-terminal His-tag cloned into a gWIZ expression vector was available from a previous study (Göritzer et al., 2019).

### 2.6 Recombinant protein production

Cultures of Agrobacteria containing the heavy and light chain of COVA2-15 IgA were set up and introduced into 6-week-old *N. benthamiana* wildtype, hence referred to as “WT”-produced IgA1, or T2 plants of the NbP4H10 KO-line pB109-1C, hence referred to as “H10-KO” line and “H10-KO”-produced IgA1, respectively, by syringe-mediated infiltration (Göritzer et al., 2017). Additionally, T2 plants of the H10 KO-line were co-infiltrated with *A. tumefaciens* containing vectors for expression of GalNAc-T2 and C1GalT1-glycosyltransferases to produce IgA1 (hence referred to as “H10+O” IgA1 variant) with human core1 O-glycan structures in the plant expression system (Dicker et al., 2016). Five days post infiltration plant material was harvested, and the three samples were processed separately as previously described (Göritzer et al., 2017). The monomeric IgA2 and IgG variants of the COVA2-15 antibody were produced in glycoengineered *N. benthamiana* ΔXT/FT plants (Göritzer et al., 2024).

**FIGURE 1:**
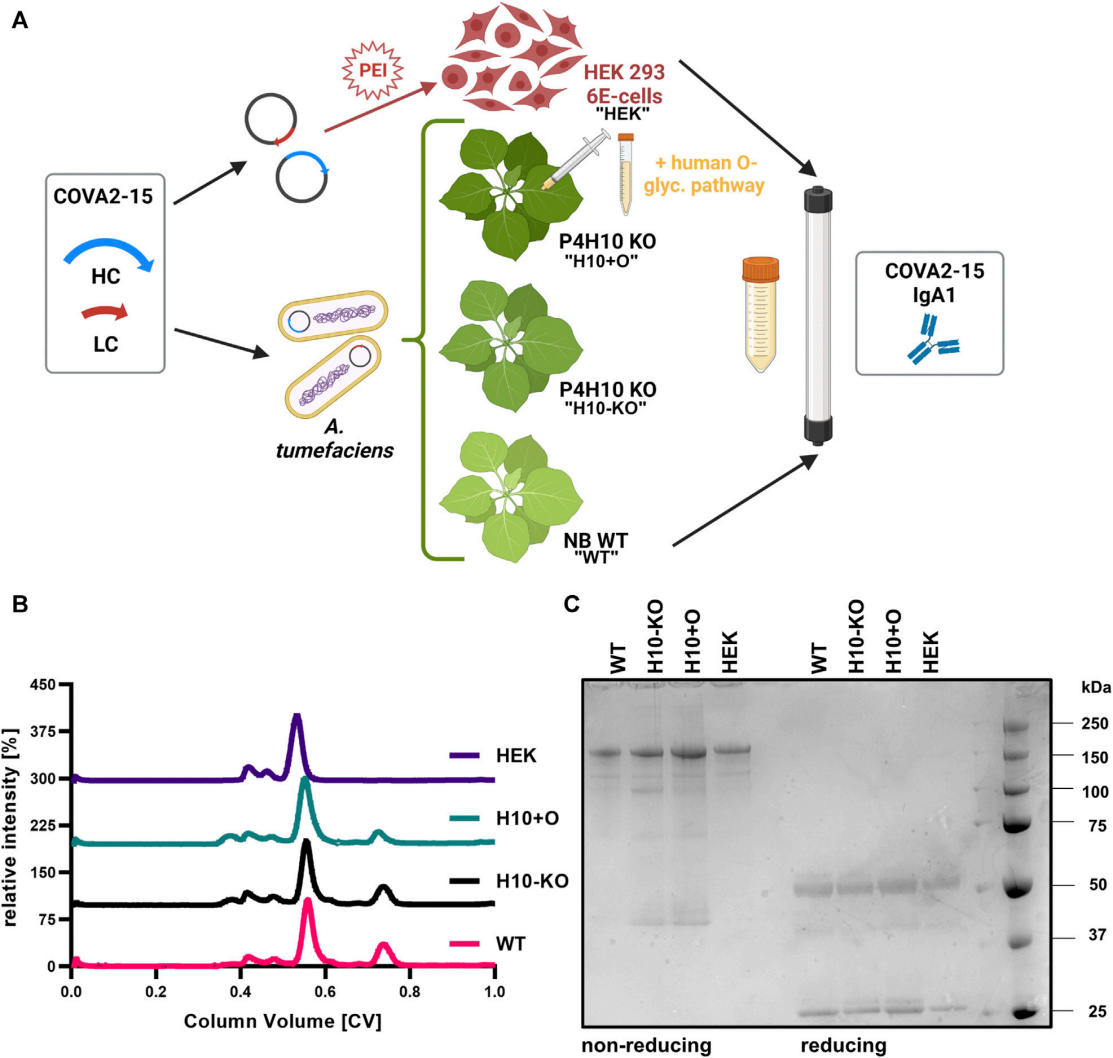
Production of recombinantly produced COVA2-15 IgA1. **(A)** Schematic illustration of transient protein expression in the different hosts and purification of four variants (“WT,” “H10-KO,” “H10+O,” “HEK”). Created with BioRender.com. **(B)** Normalized size-exclusion chromatograms of affinity-purified monomeric IgA1 COVA2-15 variants. **(C)** SDS-PAGE of affinity- and size-exclusion purified monomeric IgA1 COVA2-15 variants visualized by Coomassie Brilliant Blue staining. Samples were analysed under reducing and non-reducing conditions.

Transient expression of COVA2-15 IgA1 in HEK293-6E cells (hence referred to as “HEK”-produced IgA1) was performed according to the manufacturer’s instructions (see Figure 1A for an overview of all COVA2-15 IgA1 variants produced). Cells were cultivated and passaged routinely in FreeStyle^TM^ expression medium (Thermo Fisher Scientific, Austria). After isolation of plasmid DNA for transfection with the PureYield^TM^ Plasmid Midiprep System (Promega, Germany) transfection and harvest as well as subsequent size-exclusion chromatography (SEC) were carried out as described previously (Göritzer et al., 2017).

Recombinant CD89 was transiently expressed in Expi293F™ cells (Thermo Fisher Scientific, Austria) according to the manufacturer’s protocol. Cell culture supernatant was harvested after 7 days. CD89 was purified as previously described (Göritzer et al., 2019).

### 2.7 Mass spectrometry

Three individual biological replicates per sample were submitted for glycopeptide analysis to determine the glycosylation patterns of the recombinantly produced COVA2-15 antibody. Namely, 20 µg of purified IgA1 produced in either the *N. benthamiana* wildtype (“WT”), the T2 plants of the H10 KO-line (“H10-KO”), the T2 plants of the H10-KO co-infiltrated with GalNAc-T2 and C1GalT1 (“H10+O”) or the mammalian-system (“HEK”) were analysed (Figure 1A). The proteins were S-alkylated with iodoacetamide and digested in solution with trypsin and Glu-C (Promega, Austria).

The digested samples were loaded on a nanoEase C18 column (nanoEase M/Z HSS T3 Column, 100Å, 1.8 µm, 300 μm × 150 mm, Waters), detected with an Orbitrap MS (Exploris 480, Thermo Fisher Scientific, Austria) and the resulting glycopeptides analysed from deconvoluted spectra using protocols described previously (Billerhart et al., 2023).

### 2.8 SDS-PAGE

For non-reducing SDS-PAGE 3 µg of purified IgA1 was mixed with NuPAGE™ (Life Technologies, UK) Sample Buffer (4X) and incubated at room temperature for 5 min. For reducing SDS-PAGE 10% 2-mercaptoethanol was added to the buffer and the samples were incubated at 100°C for 5 min. The samples were loaded onto a NuPAGE™ 4%–12%, Bis-Tris gel (Life Technologies, UK) and separated proteins detected by InstantBlue (Expedeon, UK) staining.

### 2.9 Thermal shift assay (TSA)

The thermal stabilities of the four COVA2-15 IgA1 samples and the IgA2 variant were analysed by a thermal shift assay using a CFX Real-Time PCR system for detection (Bio-Rad, UK). The temperature was held for 10 s plus plate read per degree using 0.1°C increments ranging from 21°C to 95°C. Each sample was prepared and measured in triplicate, containing 10 µg of purified protein and 5 x SYPRO Orange Dye (Bio-Rad, UK) in either 25 µL H_2_O or purified protein dialyzed in a Slide-A-Lyzer MINI device (Thermo Fisher Scientific, UK) overnight at 4°C in 25 mM Na_2_HPO_4_/NaH_2_PO_4_ buffer, pH 7.4 and transferred into a standard 96-well PCR plate (Starlabs Semi-Skirted FAST, UK). The resulting curves were fitted using a Boltzmann Equation to identify the melting temperature (Tm) (Huynh and Partch, 2015).

### 2.10 Stability in human saliva and BAL fluid

A healthy volunteer that had not been infected with SARS-CoV-2 but received two doses of vaccination donated saliva for this study. The saliva contained low levels of RBD-specific IgG (Ma, personal communication) and was stored at −20°C before being processed as described before (Göritzer et al., 2024). Aliquots of the supernatant were mixed with 10 µg of each COVA2-15 variant (IgA1s, IgA2, and IgG, respectively), and samples collected immediately (timepoint 0) as well as after incubation at 37°C after 1, 2, 4, 24, 48, and 168 h, respectively.

Bronchoalveolar lavage (BAL) fluid pooled from several transgenic BALB/c mice was available from a previous study (Tran et al., 2020) and stored at −20°C. Immediately before usage, BAL fluid was thawed and centrifuged at 3000 x g for 10 min. The supernatant was then concentrated 10 x using Amicon centrifugal filters (10 kDa MWCO, Merck Millipore, Germany). Samples were prepared as described above for saliva and aliquots collected immediately (timepoint 0) as well as after incubation at 37°C for 1, 4, 24, 48, 72, and 168 h.

A sandwich ELISA was performed as described previously (Göritzer et al., 2024) to determine the proteolytic stability of the antibodies in both human saliva and transgenic BALB/c mice BAL fluid. Samples were diluted in blocking solution to 0.1 μg/mL and 0.5 μg/mL, respectively, and added to the wells in normalized concentrations. For IgA variants HRP-conjugated goat anti-human IgA alpha chain (ab97215, Abcam, UK) and for IgG HRP-conjugated goat anti-human IgG-Fc (AP113P, Merck, Germany) were used for detection. Wherever applicable, a one-phase decay non-linear model was used to calculate half-lives of the antibodies in the fluids.

### 2.11 Proteolytic stability

To estimate the influence of hinge-region O-glycans on the antibodies’ proteolytic stability 5 µg of each mIgA1 variant was incubated with 5 μg/mL of purified meningococcal IgA1 protease (Besbes et al., 2015) in a total of 50 µL PBS buffer and incubated for 6 h at 37°C. Control samples without the addition of protease were treated similarly. The reaction was stopped by adding 1x Laemmli buffer and boiling the samples for 5 min. The reduced samples were loaded on a 4%–12% SDS-PAGE gel, and upon separation, the proteins were transferred to nitrocellulose membranes. The membranes were incubated with goat anti-human IgA (alpha) peroxidase-conjugated antibody (R&D Systems, USA) at a dilution of 1:5000 and developed using Pierce ECL Western Blotting Substrate (Thermo Fisher Scientific, UK).

ELISA assays were carried out to quantify the protease activity in each sample. The plates were coated overnight with 5 μg/mL of IgA1 kappa antibody at room temperature. Coated wells were incubated with the COVA2-15 IgA1 variants at 5 μg/mL for 16–18 h at room temperature. After 5 washes with PBST serial dilutions of the meningococcal IgA1 protease (0–10 μg/mL) were applied to the wells. Following incubation for 6 h the plates were washed 5 times and blocked for 1 h at 37°C with PBST containing 0.5% BSA (w/v). The undigested antibodies were detected with goat anti-human IgA (alpha) peroxidase-labelled antibody (R&D Systems, USA) at a dilution of 1:5000 and the protease activity read out via absorbance at 492 nm on a plate reader. The relative activity was expressed as the ratio of digested samples to undigested controls.

### 2.12 Virus neutralisation assay

The pseudo-virus neutralisation experiment was carried out as described previously (Göritzer et al., 2024). Purified antibodies (COVA2-15 IgA1 and IgA2 variants, COVA2-15 IgG) were sterile filtered and serially diluted starting at 100 μg/mL.

### 2.13 Cell lines and cell culture

U937 cells (human monocyte cell line) were obtained from ATCC (LGC Standards, UK) and maintained in complete Roswell Park Memorial Institute (RPMI) – 1640 Medium (Gibco, UK) containing 10% fetal bovine serum, 5 mM L-glutamine, 100 U/mL penicillin, 100 μg/mL streptomycin, and 10 mM (4-(2-hydroxyethyl)-1-piperazineethanosulfonic acid (HEPES) buffer (all Sigma-Aldrich, UK) in a humidified CO_2_ incubator (5% CO_2_ and 37 °C). U937 cells were grown and propagated in T75 flasks (Falcon, UK). Cells were maintained at 2-3 million cells/mL density. Only cultures with at least 90% viability were used in experiments. Cell viability and cell counts were obtained using trypan blue exclusion method wherein equal volumes of cells and 0.4% trypan blue (Bio-Rad, UK) were mixed, placed on counting slides (Bio-Rad, UK), then counted using an automated cell counter (TC20, Bio-Rad, UK).

### 2.14 Surface plasmon resonance (SPR) spectroscopy

The kinetic parameters of the binding interaction between recombinantly produced monomeric COVA2-15 IgA variants with the ligands SARS-CoV-2 RBD-His and CD89 were estimated on a BIAcore X-100 instrument (GE Healthcare, UK) at 25°C. The CM5 chip was prepared, and parameters were set as described previously (Göritzer et al., 2024). RBD-His was diluted to a concentration of 4 μg/mL for the first technical replicate and 1 μg/mL for the second one. This was necessary due to batch-to-batch variation in the RBD preparations leading to different binding interactions during the experiment. The CD89 ligand was consistently diluted to 1 μg/mL in HBS-EP+ buffer. BIAcore Evaluation software was used and a 1:1 Langmuir model of binding was employed to fit blanked sensorgrams. Duplicate measurements were performed for all samples and reactants, respectively.

### 2.15 *In vitro* CD89-mediated cell binding assay of COVA2-15 IgAs

*In vitro* CD89 (FcαRI) receptor-mediated binding capacity of the COVA2-15 IgA variants was studied by using cell line U937 in comparison to a human serum IgA control. To ensure that binding to cells was specifically CD89-mediated, in some assays we pre-incubated the different antibodies as well as a human serum IgA control with 31 ng/μL soluble CD89 at 37°C for 1 hour. About 5 × 10^5^ U937 cells were added to a U-bottom 96-well plate and washed twice with sterile PBS. Afterward, 200 µL of control (PBS), human serum IgA (100 μg/mL) (Sigma-Aldrich, UK) or COVA2-15 IgA samples (100 μg/mL), as well as the pre-incubated antibody samples with soluble CD89, was added and cells were incubated in the fridge for 90 min. Cells were washed with 3 mL sterile PBS, and then a cocktail of live/dead stain and/or goat anti-human IgA-FITC (Sigma-Aldrich, UK) was added. Cells were incubated for 30 min in the fridge and washed with 3 mL sterile PBS.

### 2.16 CD14-positive monocyte isolation

CD14^+^ monocytes were enriched from PBMCs using the MojoSort^TM^ Human Pan Monocyte isolation kit (BioLegend, UK) according to the manufacturer’s recommendations. Monocyte isolation yielded >90% purity and >95% viability of CD14^+^ monocytes.

### 2.17 Cell internalization assays of COVA2-15 IgA RBD-VLP complexes

We assessed the uptake of IgA-virus-like particle (VLP) complexes by CD14^+^ monocytes via phagocytosis both quantitatively and qualitatively. For this experiment, we included a human serum IgA as a control, the four monomeric IgA1 variants described previously, and two additional ones produced in the *N. benthamiana* ΔXT/FT plants (Strasser et al., 2008): a monomeric COVA2-15 IgA1 (“DXF”) and secretory IgA (“SIgA”). VLP-displaying SARS-CoV-2 RBD (SARS-CoV-2 RBD-VLPs) were kindly provided by George Lomonossoff (Jung et al., 2022). Antibody-VLP complexes were made by incubation of 50 µL antibodies (10 μg/mL) with 5 × 10^4^ RBD-VLP particles for 2 h at room temperature. The VLPs were previously analysed by FACS and the batch was estimated to contain around 5000 particles/µL.

For flow cytometry experiments, 100,000 cells/well were seeded in a 96-well plate in 100 µL medium and treated with 100 µL of either control (PBS), antibody-VLP complexes (10 μg/mL), or human serum IgA (10 μg/mL) and RBD-VLPs alone and incubated for 3 h at 37°C. RBD-VLP-positive monocytes were counted with FACS.

For microscopy experiments, cells were plated in 96-well black plates (Corning Inc., UK). Antibody-VLP complexes were then incubated with cells for 3 h. Afterward, wells were gently washed with PBS, followed by fixation and permeabilization for 15 min. Intracellular staining for both F-actin and IgA was done using 1:2000 diluted Phalloid-iFluor647 (ab176759, Abcam, UK) and 1:100 diluted goat anti-human IgA-FITC (Sigma-Aldrich, UK), respectively, for 45 min in 4°C. Finally, nuclear counterstaining with 1:1000 diluted DAPI (Thermo Fisher Scientific, UK) for 5 min was done. Stained cells were resuspended in PBS before imaging using a NikonA1R confocal microscope. Image acquisition was carried out through Nikon NIS-Elements C software followed by image generation using NIS Elements (Nikon, UK).

### 2.18 Monocyte-derived dendritic cell (moDC) differentiation and activation

Enriched CD14^+^ monocytes were plated on 24-well tissue culture plates (Corning Inc., UK) at a density of 1 × 10^6^ cells per well. Complete RPMI 1640 media supplemented with 70 ng/mL granulocyte-macrophage colony-stimulating factor (GM-CSF) (Peprotech, UK) and 50 ng/mL interleukin-4 (IL-4) (BioLegend, UK) was used for 7 days to induce differentiation of monocytes to monocyte-derived dendritic cells (moDCs). Fresh differentiation media was added to wells every 2 days. At day 7, cells were detached from wells using an enzyme-free dissociation solution (Merck, UK) and re-plated at a density of 5 × 10^4^ cells/well in 96-well U bottom tissue culture plates (Corning Inc., UK) in complete RPMI-1640.

The four monomeric COVA2-15 IgA variants as well as two samples produced in the *N. benthamiana* ΔXT/FT plants (Strasser et al., 2008), namely the monomeric COVA2-15 IgA1 (“DXF”) and secretory IgA1 (“SIgA”), were incubated with RBD at 4 x molar ratios for 2 hours at room temperature to form complexes since it was reported that dendritic cells get activated primarily by immune complexes. Additionally, a control containing only RBD (20 μg/mL) was incubated alongside the antibody samples. Cells were subsequently treated in triplicates with either lipopolysaccharide (LPS) (100 ng/mL) (Sigma-Aldrich, UK), IgA1 variants, or SIgA1 complexed with RBD (10 μg/mL), or RBD alone (10 μg/mL). Untreated cells in triplicate were used as FMO. At day 9, cells were harvested to measure activation-induced cell-surface markers (MHC-I/HLA-ABC, MHC-II/HLA-DR, CD80, CD86, and PD-L1) through flow cytometry. Briefly, wells were washed with DPBS (Sigma-Aldrich, UK) and then incubated for 45 min in 4°C with 1:500 bioscience fixable viability dye eFluor780^TM^ (Invitrogen, UK), 1:250 Human TruStain FcX^TM^ (BioLegend, UK), 1:200 APC anti-human HLA-DR (clone L243, UK), 1:200 PE anti-human HLA-A, B, C (clone W6/32, UK), 1:200 PE/Cyanine 7 anti-human CD86 (clone IT2.2, UK), 1:200 Brilliant Violet 510^TM^ anti-human CD80 (clone 2D10, UK), and 1:200 Brilliant Violet 42^TM^ anti-human PD-L1 (clone 29E.2A3, UK) in DPBS. After incubation, cells were washed and resuspended in DPBS until FACS analysis.

### 2.19 Flow cytometry and data analysis

Stained cells were acquired using CytoFLEX S (Beckman Coulter, UK) flow cytometer. Files were exported and analysed using FlowJo^TM^ v10.8.1 (TreeStar, UK). For all experiments, at least 5000–10000 events were obtained per sample. Gating strategies are shown in Supplementary Figure S1.

### 2.20 Statistical analyses

All statistical analyses were carried out using GraphPad Prism 9. We used a cut-off of 5% for the adjusted *p*-value for multiple comparisons in a two-way ANOVA in the CD89-mediated cell-binding assay, an ordinary one-way ANOVA for the CD14^+^ monocyte uptake, an ordinary one-way ANOVA for the dendritic cell activation assay.

## 3 Results

### 3.1 Generation of stable *NbP4H10* double mutant lines

Based on our previous findings regarding the implications of *P4H* subfamilies on O-glycosylation in *N. benthamiana* and transcriptome data (Gene Expression Atlas v6, https://benthgenome.qut.edu.au/) suggesting high activity in leaf tissues, we selected *NbP4H10* as genome editing target in our study (Mócsai et al., 2021; Uetz et al., 2022). To identify P4H10 paralogs in *N. benthamiana* a BLAST search was performed using the previously established nucleic acid and protein sequences of gene Nbv6.1trP31841 (Queensland University of Technology database annotation, herein referred to as *NbP4H10_2*) as a query against available *N. benthamiana* databases. The respective gene was annotated in the other *N. benthamiana* sequence databases and another putative *NbP4H10* gene (*NbP4H10_1*, Supplementary Table S1), which shared high sequence identity with *NbP4H10_2,* was identified. These two initially identified genes were targeted by genome editing. A later discovered additional *NbP4H10* gene (*NbP4H10_3*) was not targeted by the approach.

After aligning the sequences of the two *NbP4H10* genes (*NbP4H10_1* and *NbP4H10_2*), we identified 8 pan-specific gRNAs (G1-G8, Supplementary Table S3) targeting both without any predicted sequence homologous off-target effects. The gRNAs were inserted into binary plant transformation plasmids and transiently expressed in wildtype *N. benthamiana* plants to estimate gRNA editing efficiencies as described previously (Uetz et al., 2022). Remarkably, all gRNAs induced mutations (Supplementary Table S4), causing one bp insertions or deletions (INDELs) at efficiencies ranging from 8.1% (G1 targeting *NbP4H10_1*) to 48.4% (G4 targeting *NbP4H10_2*). The final plant transformation vector contained guides G3 and G7 displaying the highest overall INDEL scores (39.4% and 42.3% for *NP4H10_1*% and 34.9% and 35.8% for *NP4H10_2*, respectively).

In total, we recovered 13 viable T0 plants carrying the Cas9 transgene as evidenced by PCR screening. All but three plants also displayed genome-editing events at the two target genes (Supplementary Table S5). By the transient assay, most mutations were one or two bp INDELs apart from some bigger deletions ranging from 5 to 8 bp. Plants NbpB109-1A, -1B, -1C, −6, and −10 showed high editing efficiencies at both target sites (reaching up to 99% for G7 in NbpB109-1A) and were thus considered suitable candidates for establishing a stable line retaining the mutations over following generations. However, when assaying 30–50 plants of each of the T1 generation that were obtained after self-pollination of T0 plants, we could identify Cas9-negative segregants only in the NbpB109-1C line (referred to as “H10-KO”-line). No visual phenotype was observed for any of the T1 plant lines or in further generations.

### 3.2 Purification of monomeric anti-SARS-CoV-2 IgA1 antibodies

To test the impact of functional knock-out of two *P4H10* genes on O-glycosylation of a recombinant glycoprotein, human IgA1 was produced and characterized. Four SARS-CoV-2 specific (COVA2-15) monomeric IgA1 variants were recombinantly produced in either *N. benthamiana* WT (“WT”), H10-KO plants (“H10-KO”), H10-KO plants co-infiltrated with *A. tumefaciens* containing vectors for expression of human GalNAc-T2 and *Drosophila melanogaster* C1GalT1 glycosyltransferases (“H10+O”) previously used for IgA1 O-glycan modifications (Dicker et al., 2016), and a HEK293-6E cell expression system (“HEK”) (Figure 1A). Additionally, IgA2 and IgG1 variants of this antibody were transiently expressed in glycoengineered *N. benthamiana* ΔXT/FT plants (Strasser et al., 2008). Following affinity purification of all antibodies, we performed size-exclusion chromatography (SEC). All COVA2-15 IgA1 SEC profiles showed a major peak at a retention time indicative of the monomeric form of the antibody while containing minor peaks at lower retention times that likely contain high molecular weight aggregates (Figure 1B). Additionally, a minor non-characterized peak at higher retention times was observed for the plant-produced variants (WT, H10-KO, H10+O, respectively) that is absent in the mammalian-produced sample and represents free heavy chain or degradation products. When resolving the SEC fractions containing the monomeric IgA1 variants under non-reducing conditions on an SDS-PAGE gel (Figure 1C), a major band was detected at a molecular mass around 160–170 kDa indicative of the fully assembled monomer. The band for the HEK IgA1 variant appears at a higher molecular weight, potentially due to glycosylation with more complex N-glycans. Lesser amounts of additional bands are visible in the lanes for the H10-KO and glycoengineered mutant, which are not as prominent under reducing conditions, where a band representing the heavy chain at around 55 kDa and the light chain at around 25 kDa can be observed.

### 3.3 Plant-specific O-glycans are reduced on recombinant IgA1 antibody produced in the *P4H10*-KO

**FIGURE 2:**
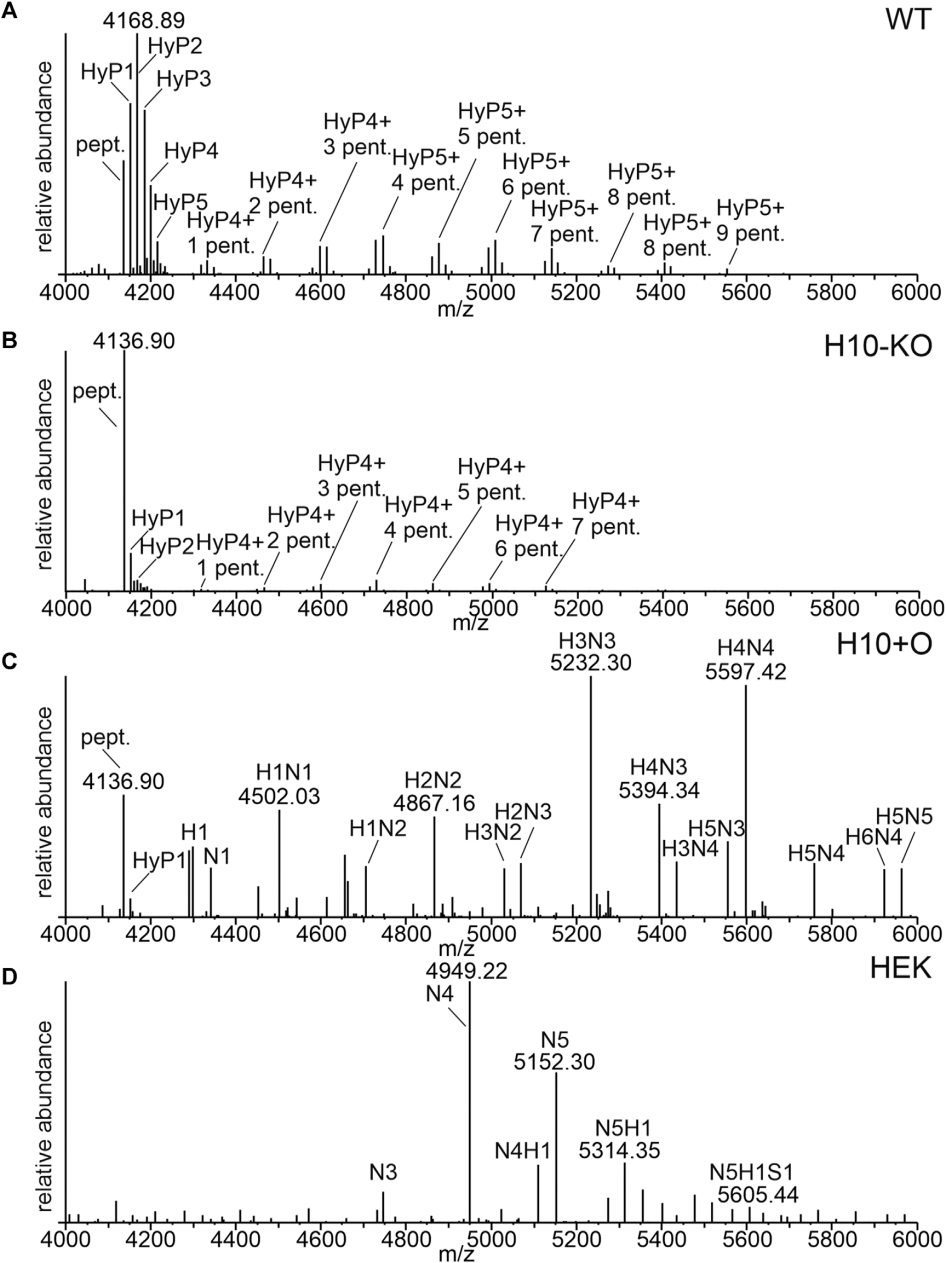
O-glycosylation profiles of recombinantly produced COVA2-15 monomeric IgA1 variants. **(A)** LC-ESI-MS spectrum of the hinge region peptide from COVA2-15 IgA1 produced in WT. The unmodified peptide (pept., HYTNPSQDVTVPCPVPSTPPTPSPSTPPTPSPSCCHPR, 4136.89 [M+H]^+^), hydroxyprolines (HyP) and pentoses (pent.) are indicated. **(B)** MS-spectrum of the hinge region from COVA2-15 IgA1 produced in H10-KO. **(C)** MS-spectrum of the hinge region peptide from COVA2-15 IgA1 produced in H10-KO co-infiltrated with the human core-1-O-glycosylation pathway. H indicates hexoses, and N indicates N-acetylhexosamine. **(D)** MS-spectrum of the hinge region peptide from HEK293-6E cell produced COVA2-15 IgA1. S indicates N-acetylneuraminic acid.

The glycosylation of the IgA1 hinge region of the COVA2-15 antibody was analysed by LC-ESI-MS and compared between wild-type-produced (“WT”) and “H10-KO” produced IgA1 (Figures 2A, B). We observed up to six proline residues that were hydroxylated in the WT (Supplementary Table S6), with varying amounts of additional pentoses that represent arabinose chains. By contrast, the spectrum for the H10-KO-produced variant displayed reduced amounts of hydroxylated and glycosylated peptides (Figure 2B). For quantitative analysis of the relative abundance of HyP formation in WT and H10-KO-produced IgA1, peak integration was performed. An increase in relative amounts of unmodified hinge-region peptide could be detected between the mutant (55.1%) and wild-type (8.2%). The H10-KO-produced IgA1 hinge-region peptide contained hydroxylated moieties (mainly HyP1 and HyP2) and harboured small amounts of peptide further modified with pentose residues (Supplementary Table S6). This finding provides evidence that the *NbP4H10* subfamily catalyses HyP formation on recombinant IgA1 and because of abolished HyP formation, the transfer of pentoses by the plant-endogenous O-glycosylation pathway is affected.

COVA2-15 IgA1 H10+O, which was obtained by co-expression of GalNAc-T2 and C1GalT1 in the H10-KO line displayed a mixture of residual plant-type O-glycan moieties such as HyP and human mucin-type core 1 O-glycans (Figure 2C). HEK-produced IgA1 displayed less of the unmodified hinge region peptide and HexNAc modifications with low amounts of sialylated O-glycans (N5H1S1 labelled peak, Figure 2D). While the HEK IgA1variant is virtually fully glycosylated harbouring predominantly HexNAc4 and HexNAc5 decorated residues (Supplementary Table S7), the H10+O sample showed a broad spectrum of O-glycosylated hinge-region O-peptides, containing among Hex3HexNAc3- and Hex4HexNAc4-modified residues a considerable amount of unmodified peptide (around 9%). Due to the heterogeneity of O-glycoforms present in the hinge region of IgA1 when produced in mammalian cells and mutant plants with co-expression of heterologous O-glycosylation enzymes, the assignment of peaks to specific amino acid modifications in the hinge region was not possible.

No changes in the N-glycan profile between any of the plant-produced samples could be detected (Supplementary Figure S2) which is consistent with previous data showing no interference with other glycosylation pathways (Castilho et al., 2012; Dicker et al., 2016).

### 3.4 Glycoengineered COVA2-15 IgA1 antibodies neutralize SARS-COV-2

**FIGURE 3:**
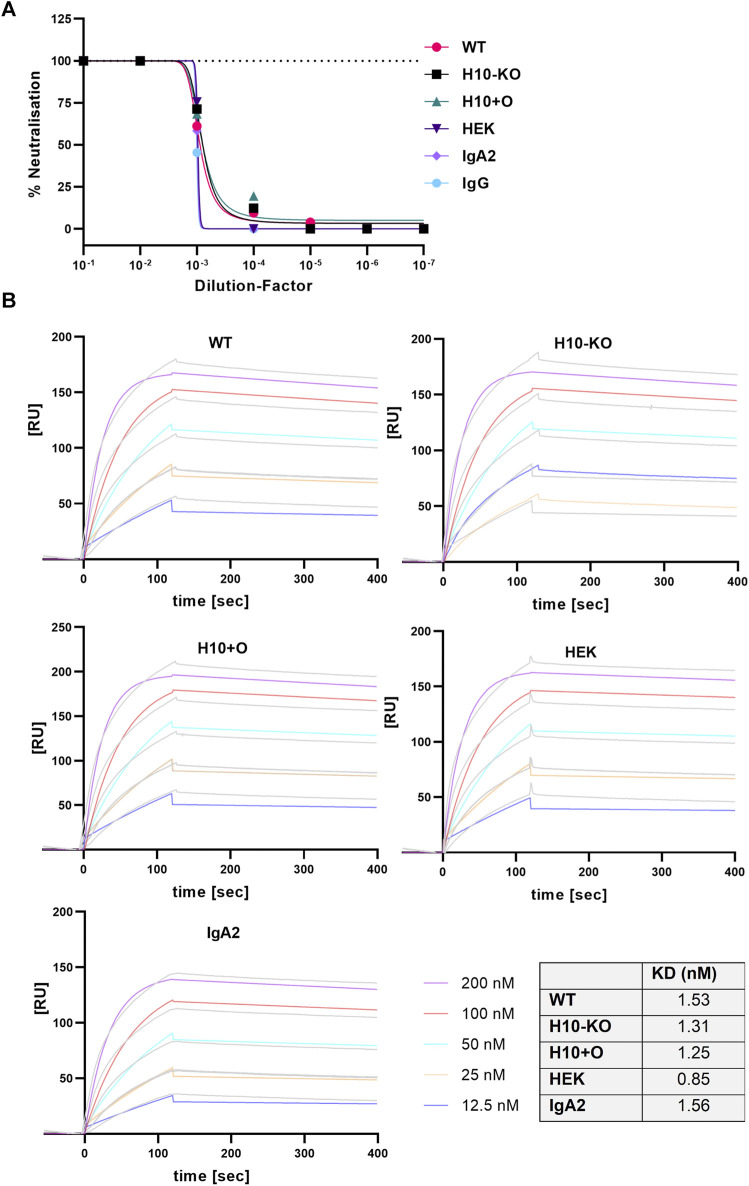
SARS-CoV-2 virus neutralisation and RBD-binding of COVA2-15 antibodies. **(A)** Neutralisation of SARS-CoV-2 (England 02/2020) by COVA2-15 IgA1 variants. Neutralisation capacity was measured using a PRNT assay on Vero E6 cells, COVA2-15 IgA2 and IgG were included as controls. MAbs were added in serial 1:10 dilutions starting with 100 μg/mL. The mean of duplicates of one representative out of two experiments with comparable results is shown. **(B)** Binding kinetics of COVA2-15 IgA1 variants to RBD obtained by SPR spectroscopy in multi-cycle kinetic experiments. IgA2 was included as a control. Five different concentrations of the respective mAb were injected. The obtained curves were fitted with a 1:1 binding model. Data shown are from one experiment representative of at least two technical repeats.

To test the capability of the four IgA1 variants to neutralize a clinical isolate of SARS-COV-2 (England/2/2020) we performed a plaque reduction assay in Vero E6 cells stably expressing the human cell receptor ACE2 and the transmembrane serine protease TMPRSS2, both playing a central role in virus entry into host cells (Hoffmann et al., 2020). Additionally, we included the IgG and IgA2 variants of the same antibody as controls. At a concentration of 0.1 μg/mL, all antibodies showed a neutralisation potency of at least 50% (Figure 3A) and the IC50 values were in a similar range (Supplementary Table S8).

### 3.5 Glycoengineered COVA2-15 IgA1 antibodies display comparable binding to SARS-CoV-2-RBD and the Fc-alpha receptor

We tested the binding kinetics of IgA1 variants to either the SARS-CoV-2 RBD (the portion of the viral spike protein that binds to the ACE2 receptor) or the specific Fc-alpha receptor (CD89) by surface plasmon resonance (SPR) spectroscopy. Both ligand proteins contained a His-tag which allowed capture onto a CM5 chip via an anti-His antibody. The COVA2-15 antibodies were injected at varying concentrations in multi-cycle experimental runs and the curves were fitted employing a 1:1 binding model. The association rates (ka) for the RBD ligand observed across all COVA2-15 mAb variants were in a similar range as the rate constants reported for COVA2-15 IgG and secretory IgA (Göritzer et al., 2024); the very low dissociation rates (kd) corresponded well to the already published values (Figure 3B).

**FIGURE 4:**
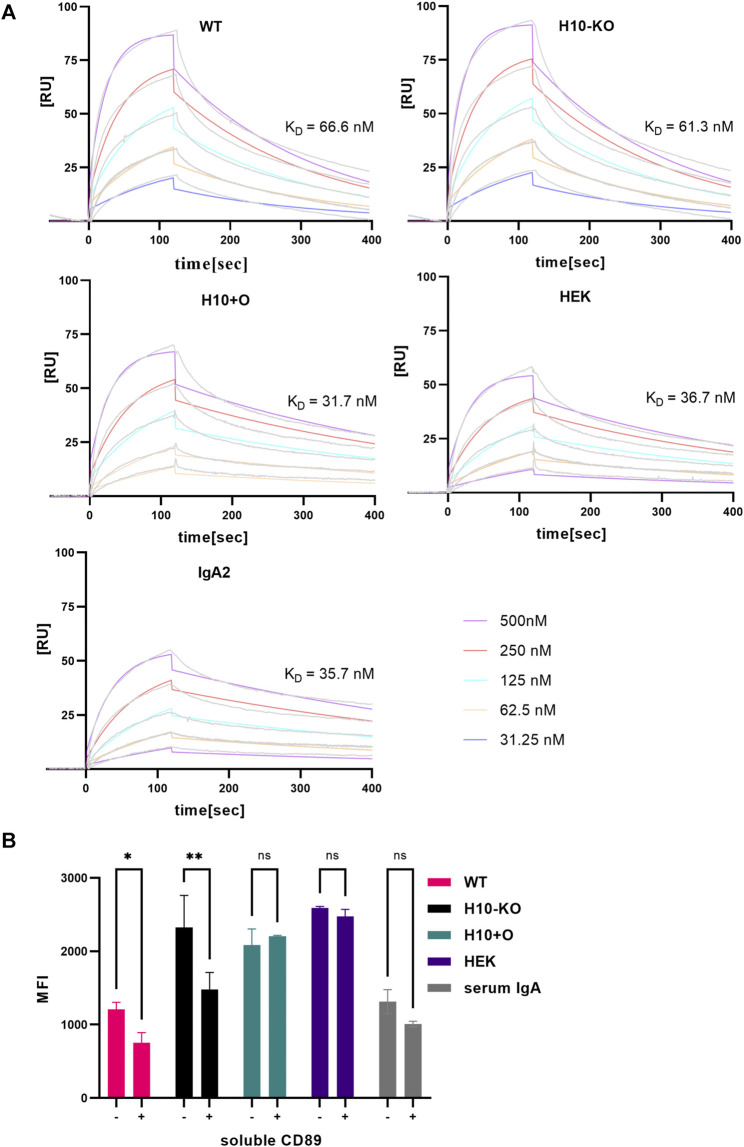
CD89 receptor-binding capacity of COVA2-15 IgA1 variants. **(A)** Binding kinetics of COVA2-15 IgA1 variants to CD89 obtained by SPR spectroscopy in multi-cycle kinetic experiments. IgA2 was included as a control. An anti-His antibody was immobilized on a CM5 chip, CD89 was captured and 5 different concentrations of the respective mAb were injected. The K_D_ values [nM] were calculated by fitting obtained curves with a 1:1 binding model and representative values of at least two technical replicates are depicted. **(B)** CD89-mediated cell binding capacity of COVA2-15 IgA1 variants. U937 cells were incubated with the IgA1 variants or a serum control and analysed using a flow cytometer. To assess the specificity of the binding the same samples were incubated with 31 μg/mL soluble CD89 before being added to the cells. Samples were measured in triplicate and the means of the same samples with (+) and without (−) soluble CD89 were used for performing a 2-way ANOVA with multiple comparisons setting the cut-off at 5% for the adjusted *p*-value (ns, not significant; *, *p* < 0.05; **, *p* < 0.01).

Overall, the affinity (K_D_) of all mAb variants to RBD was comparable to the one observed for COVA2-15 IgG (Göritzer et al., 2024), showing that the presence of distinct O-glycan modifications does not affect the antibodies’ function. For the CD89 interaction, fast association and dissociation rates were detected for all IgA1 variants (Figure 4A). The IgA2 displayed decreased association and dissociation rates compared with IgA1 across all experiments which is consistent with previous findings. The K_D_ values observed for the H10+O IgA1 and the HEK IgA1 were reduced in comparison to the WT and H10-KO IgA1, hinting at possible albeit small influence of mucin-type O-glycans on receptor binding kinetics.

### 3.6 Glycoengineered COVA2-15 IgA1 antibodies display differences in binding to CD89-expressing pro-monocytes

The pro-monocytic lymphoma cell line U937 was used to analyse the *in vitro* binding of COVA2-15 IgA1 variants to CD89. While the interaction of WT COVA2-15 IgA1 with U937 cells was comparable to the serum control, all other IgA1 variants displayed stronger binding to U937 cells, with H10+O and HEK IgA1 showing the highest overall binding (Figure 4B).

Next, we assessed the specificity of binding to monocytes via a competition assay. We incubated the IgA1 variants with soluble CD89 before adding them to the cells and found the strongest reduction in receptor binding for the H10-KO variant, while no significant effect was visible on either H10+O or HEK IgA1 that both contain mucin-type O-glycan moieties. To conclude, all COVA2-15 IgA1 variants were capable of binding to U937 cells, but in addition to FcαRI-mediated binding the binding could be mediated by lectin-type receptors and thus was not influenced by incubating with soluble CD89 (AbuSamra et al., 2022).

### 3.7 CD14^+^ monocyte uptake of IgA1-RBD-VLP complexes

To investigate the ability of the IgA1 mAbs to mediate antibody-mediated phagocytosis in CD14^+^ monocytes, immune complexes were formed by incubating recombinantly produced SARS-CoV-2 RBD-VLPs (Jung et al., 2022) with the COVA2-15 antibodies.

**FIGURE 5:**
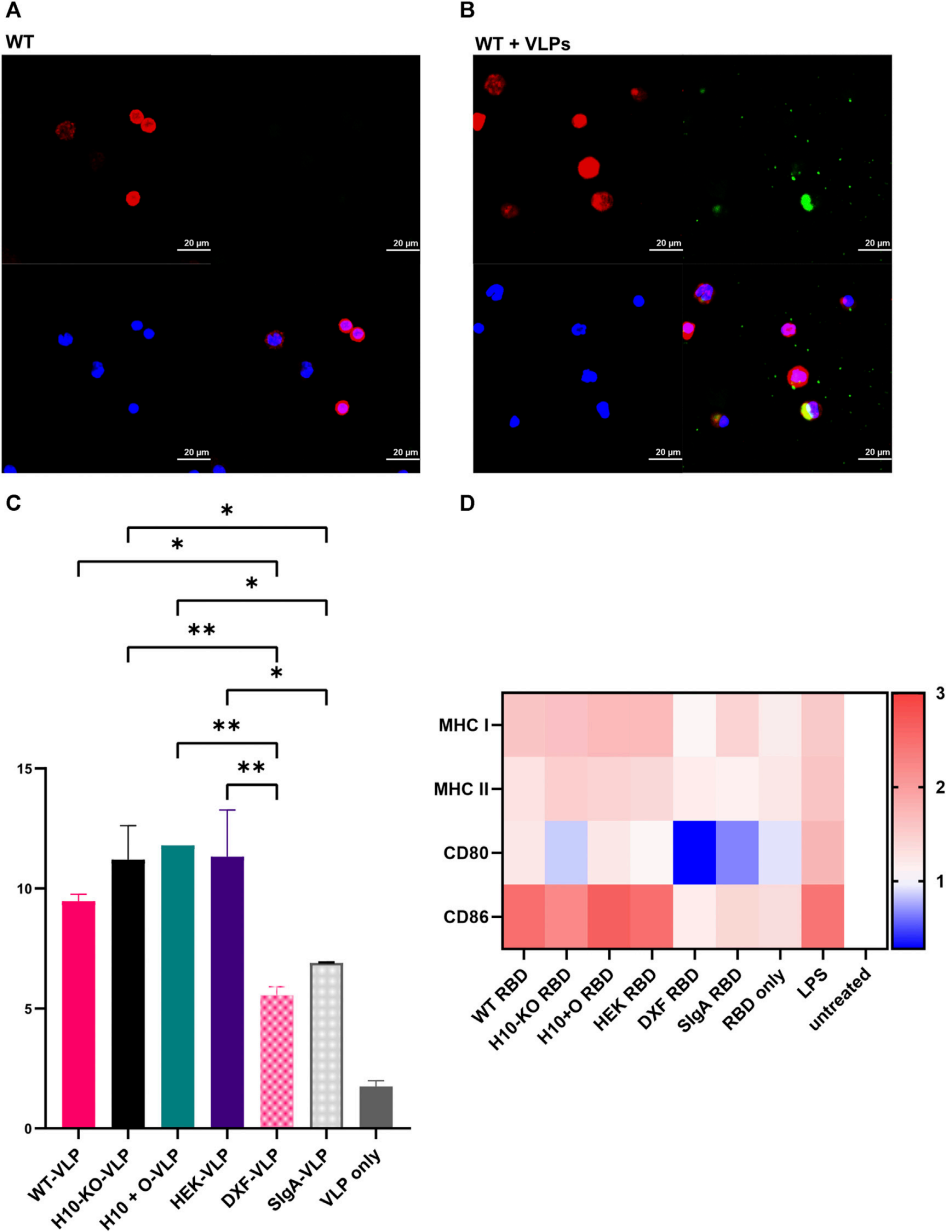
COVA2-15 IgA1 cell uptake and activation assays. **(A)** Representative confocal microscopy images depicting the antibody (WT) and **(B)** antibody-RBD-VLP (WT+VLP) uptake by CD14^+^ monocytes. Intracellular staining for F-actin (red) and IgA (green) as well as nuclear counterstaining (blue) were performed. **(C)** Antibody-RBD-VLP complexes (four IgA1 variants and three controls: VLP only, and monomeric IgA1 produced in the *N. benthamiana* ΔXT/FT plants(“DXF-VLP”) and secretory IgA (“SIgA”)) were incubated with CD14 + monocytes and RBD-VLP positive monocytes were counted with FACS. One-way ANOVA was performed on the mean of two biological replicates to compare the monocytes’ uptake of the antibody-RBD-VLP complexes. A cut-off of 5% was used for the adjusted *p*-value for multiple comparisons where the VLP-only control was not included in the analysis (*, *p* < 0.05; **, *p* < 0.01). **(D)** Heatmap representing fold-change in the expression of cell surface markers upon incubation of monocyte-derived dendritic cells (moDCs) with antibody variants (see (C)) normalized to the untreated control group. Cells were incubated with antibody-RBD complexes or the RBD alone. Additionally, LPS was used as a positive control for the stimulation of moDCs. After harvest, cells were stained with different dyes for the detection of cell surface markers (MHC I/HLA-ABC, MHC II/HLA-DR, CD80, CD86) for flow cytometry. One-way ANOVA was performed to compare the effect of antibody-RBD complexes on the RBD-only, LPS, or untreated control groups. A cut-off of 5% for the adjusted *p*-value was used for multiple comparisons.

We observed internalization of IgA1-complexed VLP in CD14^+^ monocytes in all COVA2-15 IgA1-RBD-VLP samples by confocal microscopy (Figures 5A, B). No fluorescence signal apart from ubiquitous DAPI staining and labelling of actin was detected in either the non-complexed antibody samples, the serum IgA control, or the cells treated with RBD-VLP only. Since we observed the uptake of immune complexes, we proceeded to quantification of VLP-positive monocytes via FACS (Figure 5C). There was a significant increase in the number of complex-positive cells for the samples produced in both WT, H10-KO and H10+O plants as well as HEK293-6E cells compared to those stemming from the *N. benthamiana* ΔXT/FT plants. The COVA2-15 IgA1 variants produced in WT and *N. benthamiana* ΔXT/FT plants (Strasser et al., 2008) differ in their respective N-glycan composition, with the engineered ΔXT/FT-derived sample being nearly devoid of β1,2-xylosylation and core α1,3-fucosylation. No significant influence of O-glycosylation on cell internalization could be observed between the four IgA1 variants.

### 3.8 Activation of dendritic cells (DCs) by IgA1-RBD complexes

It was shown that both the SARS-CoV-2 spike protein as well as the RBD alone can activate and mature DCs (Barreda et al., 2021), hence we used RBD as an agent to form immune-like complexes with the COVA2-15 IgA1 variants. All four IgA1 variants pre-incubated with RBD showed a significant increase of MHC I levels in DCs in comparison to both cells treated with RBD-only and the untreated control group. The upregulation of MHC I expression after treatment with the IgA1-RBD complexes was comparable to the treatment of cells with lipopolysaccharide (LPS) and thus indicative of immune stimulation (Figure 5D). MHC II levels were increased significantly only in LPS-treated DCs.

There was no significant upregulation of the co-stimulatory molecule CD80 in any of the samples, including LPS treatment, compared to levels observed in the untreated DCs. However, CD86 levels were increased significantly in DCs treated with the four IgA1-RBD complexes in comparison to both the cells treated with RBD alone or the untreated control, leading to similar levels observed after treatment with LPS. For all measured parameters, there were no significant differences in induced immune responses between any of the four IgA1-RBD complexes. The SIgA variant did not lead to any notable activation of DCs.

### 3.9 Glycoengineered IgA1 variants display differences in proteolytic stability

**FIGURE 6:**
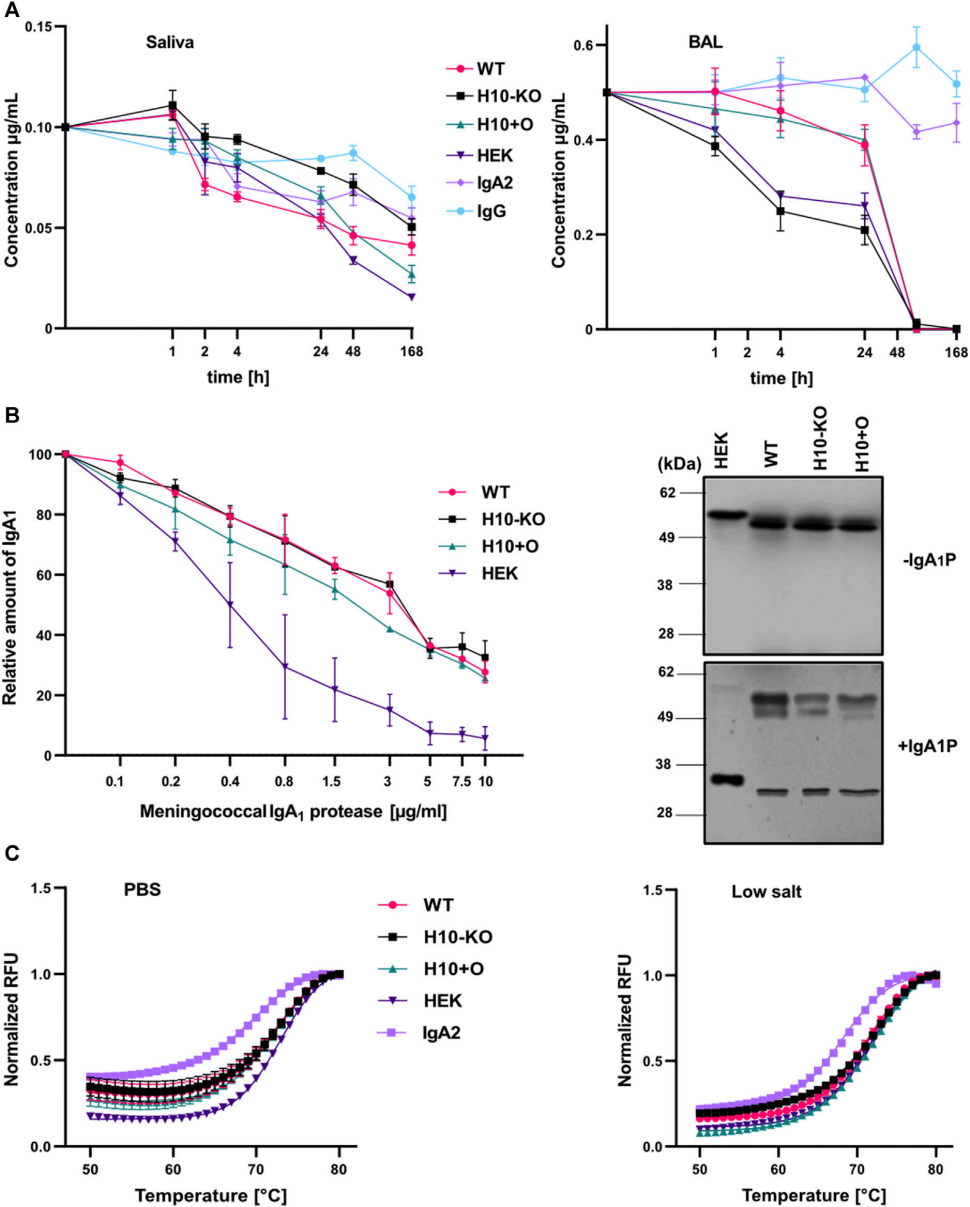
Proteolytic and thermal stability of COVA2-15 IgA1 variants. **(A)** Stability in human saliva and murine BAL (bronchoalveolar lavage) fluid. COVA2-15 IgG and IgA2 were included as controls. Saliva from a donor and pooled BAL from multiple animals were mixed with the antibodies and incubated at 37°C degrees for the specified time. Samples were analysed by ELISA for the binding to RBD. The set-up was repeated twice and representative data from one technical replicate are shown. **(B)** Susceptibility of COVA2-15 mIgA1 variants to a meningococcal IgA1 protease. Serial dilutions of the protease were incubated with the antibodies and protease activity was quantified by ELISA. The mean ± SEM of triplicates is shown. Immunoblot of IgA1 antibodies incubated with or without the protease for 6 h at 37°C. Three individual experiments were carried out and representative images are presented. **(C)** Thermal stability of COVA2-15 IgA1 variants measured using a thermal shift assay. Each antibody was measured in triplicate at temperatures ranging from 21°C to 95°C. The curves ranging from 50°C to 80°C were fitted to calculate the respective melting temperatures.

To assess the resistance of different COVA2-15 IgA1 variants against proteases in an *in vitro* setting we incubated them with human saliva or BAL fluid to imitate conditions found on mucosal tissues. Additionally, we included the IgA2 and IgG1 variants of the antibody as controls. We evaluated the degradation of the variants after incubation at 37°C for a week by assaying the functional antibody concentrations in the time course samples via SARS-CoV-2 RBD-mediated capture in a sandwich ELISA. IgG displayed the highest stability in saliva, with 35% degradation after 1 week, followed by IgA2 (Figure 6A). While we could observe a similar pattern for IgG degradation in mice BAL fluid (Figure 6A), IgA2 showed almost no degradation after 1 week. Overall, IgA1 mAbs were more stable in saliva than in mice BAL fluid, where we observed a complete degradation after 3 days. The degradation levels of the samples varied between experiments. H10-KO-produced antibody was the most stable among the IgA1 variants in saliva but was lost at a higher rate in BAL fluid than all other tested mAbs. In saliva, H10+O IgA1 showed high initial stability but degraded more after incubation for a week than H10-KO and WT-produced antibodies. Interestingly, the H10-KO IgA1 antibody proved to be the most unstable in BAL fluid while the heavily glycosylated WT and H10+O variants remained nearly intact after 24 h of incubation. We calculated the half-life of the mAbs in both fluids using a one-phase decay non-linear regression model (Göritzer et al., 2024). Half-lives of antibody variants (Supplementary Table S9) in saliva ranged from around 2.8 h for the WT IgA1 to up to 33.6 h for the H10-KO-produced IgA1. Half-lives for the IgG variant and the IgA antibodies in BAL fluid were difficult to determine as they did not plateau in the tested period, thus not fitting the proposed model.

Considering that we observed differences in proteolytic stability of the mAbs in saliva and BAL fluid, we further assessed their susceptibility to an IgA1-specific protease from *Neisseria meningitidis*. After incubating the COVA2-15 IgA1 variants with the protease, immunoblotting showed that all samples were indeed susceptible to cleavage. While the HEK293-6E cells produced variant was almost completely cleaved the heavy chains of the other samples were only partially affected (Figure 6B). No clear differences were visible between the plant-produced IgA1 antibodies. Serial dilutions of bacterial protease were then used in an ELISA and an undigested heavy chain was detected to quantify the protease activity. The ELISA data confirmed the immunoblot assay and indicated that cleavage of the sensitive forms occurred in a dose-dependent manner (Figure 6B). The data also show that the mammalian-produced IgA1 was cleaved by higher concentrations of protease whereas the three plant-produced IgA1 remained partially intact with negligible differences amongst them.

### 3.10 Differences in O-glycans do not affect the thermal stability of IgA1 variants

We assessed the thermal stability of all four IgA1 mAbs and IgA2 in two different buffer systems with high and low salt concentrations. No substantial differences in melting temperatures were observed between the IgA1 variants in PBS buffer, ranging from 72.3°C for the H10-KO derived IgA1 to 72.9°C for the HEK293-6E cells produced IgA1. IgA2 showed the lowest thermal stability of 69.3°C. Changes in thermal stability between the two buffer conditions were minimal (Figure 6C; Supplementary Table S10). However, WT IgA1 exhibited a slightly lower Tm value of 71.3°C in the low salt solution. Overall, the transition midpoint temperatures correspond well with previously published data of plant-produced anti-HER2 IgA1 and IgA2 variants (Göritzer et al., 2019) suggesting that there is no or only a negligible impact of O-glycan modifications on the thermal stability of recombinant monomeric IgA1 antibodies.

## 4 Discussion

While many advancements in the engineering of plant-endogenous N-glycosylation towards the recombinant production of biopharmaceuticals containing defined and homogenous glycans have been made, limited efforts are available on engineering of the endogenous O-glycosylation pathway. In this study, we addressed this gap via the targeted knockout of *N. benthamiana P4H10* genes involved in the first steps of this PTM. Looking at previous research conducted, targeting a specific *P4H* gene in *P. patens* has led to the removal of HyP residues on recombinantly produced EPO (Parsons et al., 2013). In *N. benthamiana*, several *P4H* genes were silenced using a transient approach (Mocsai et al., 2021) and knockout of the *P4H4* subfamily resulted in minor changes in the hinge region modifications (Uetz et al., 2022). However, this is the first report of an efficient engineering of plant-endogenous O-glycans in a higher plant used for recombinant protein production.

We used the engineered line to investigate the influence of O-glycan modifications in the hinge region of IgA1 on its biochemical and functional properties. Our data show that glycoengineering did not affect the antibodies’ function in terms of SARS-CoV-2 virus neutralisation and antigen-binding in SPR experiments. However, we noticed a difference in binding to the extracellular domain of the CD89 receptor, where mucin-type O-glycosylated H10+O and HEK COVA2-15 IgA1 exhibited lowered K_D_ values in comparison to IgA1 with plant-specific hinge-region modifications. Previous studies utilizing SPR have shown that N-glycan variations in the CH2-domain of monomeric IgA1 do not influence binding to CD89 regardless of the proximity to the binding site (Gomes et al., 2008; Göritzer et al., 2019). Considering that the O-glycosylation sites of IgA1 are located at a 20–30 Å distance from the antigen-binding site one would not expect a strong influence on the interaction. Yet, we observed an influence of mucin-type O-glycans on the receptor-binding kinetic, hinting at conformational changes in the IgA1 structure.

Observing the minor differences in CD89 binding kinetics between the IgA1 variants using SPR, we hypothesized that there could be an even more nuanced variation when using a cell-based assay. It has been shown previously that glycosylation, specifically the removal of N-linked oligosaccharides of the FcαRI and the sialyation of IgA1 purified from human serum, heavily influenced cell binding (Basset et al., 1999; Xue et al., 2010). Furthermore, aberrantly glycosylated IgA1 with a galactose-deficient hinge region of patients with IgA nephropathy, bound stronger to a murine cell line than IgA1 from healthy subjects, indicating a role of glycans in the binding (Van Zandbergen et al., 1998). Alongside many other myeloid cells in human tissues (Monteiro & Van De Winkel, 2003), monocytes express the heavily glycosylated CD89/FcαRI on their surface making them a suitable model to study potential influences of the IgA1 hinge region O-glycosylation on receptor-mediated cell binding. While all produced IgA1 variants were functional in binding to the pro-monocytic cells, we observed stronger binding to U937 cells for the mucin-type O-glycan decorated HEK IgA1 and H10+O. However, preincubation of IgA1 mAbs with soluble CD89 only partially reduced cell-binding of H10-KO IgA1. There were no distinct changes in the mucin-type O-glycosylated IgA1 variants, which indicates that binding to U937 cells was mediated by other lectin-type receptors (AbuSamra et al., 2022).

Since it is known that IgA immunocomplexes bind avidly to and cross-link FcαRI leading to phagocytosis and cell uptake, we wanted to assess the phagocytotic capacities of our antibody variants complexed with RBD-VLPs by CD14^+^ monocytes (van Egmond et al., 2000; Wines et al., 2001; Herr et al., 2003; Oortwijn et al., 2007; Breedveld and Van Egmond, 2019). SIgA was previously reported to not induce phagocytosis in both neutrophils and Kupffer cells (van Egmond et al., 2000; Vidarsson et al., 2001) and was included as a control. Interestingly, we observed the uptake of all IgA1-RBD-VLP complexes including SIgA1 by CD14^+^ monocytes. To quantitatively assess the cell internalization of immune complexes we performed FACS and found that there was no significant difference in CD14^+^ monocyte uptake between complexed WT H10, H10+O, and HEK COVA2-15 IgA1. However, there were less complex-positive cells detected for the IgA1 produced in *N. benthamiana* ΔXT/FT plants (Strasser et al., 2008), which contain modified N-glycan structures. In accordance with a previous study (Gomes et al., 2008), we conclude that phagocytosis of monocytes is regulated to some degree by antibody N-glycosylation, but not O-glycosylation.

Monocyte-derived dendritic cells (moDCs) are very efficient antigen-presenting cells (APCs) involved in pathogen recognition and induce a plethora of immune responses including the secretion of cytokines and lipid mediators (van Vliet et al., 2007). Like most other cells of myeloid origin, they express CD89/FcαRI and several other IgA-binding receptors such as the transferrin receptor, DC-SIGN, and Dectin-1 on their surface (Gayet et al., 2020). Upon binding of IgA-antigen complexes, DCs are activated as evidenced by upregulation of class II and class I MHC and costimulatory molecules such as CD80 and CD86, leading to DC maturation (Banchereau and Steinman, 1998; Geissmann et al., 2001; Janeway and Medzhitov, 2002). Therefore, we wanted to assess the potential of the COVA2-15 IgA1 mAbs to provoke a pro-inflammatory response leading to maturation of moDCs. It was previously shown that RBD of SARS-CoV-2 can efficiently activate and mature DCs (Barreda et al., 2021), hence we used this antigen as a complexing agent for the antibodies under study. While we did observe an increase in both MHC II and MHC I levels for moDCs incubated with COVA2-15 IgA1-RBD complexes, only MHC I expression increased substantially. Interestingly, the *N. benthamiana* ΔXT/FT (Strasser et al., 2008) produced IgA1 did not induce MHC I expression. While Barreda et al. (2021), found RBD to induce upregulation of both CD80 and CD86 levels in moDCs, we did not observe significant overexpression of CD80 molecules after treatment with IgA1-RBD complexes but did observe a substantial increase for CD86 levels by all COVA2-15 IgA1s, except *N. benthamiana* ΔXT/FT produced IgA1. SIgA did not lead to any notable activation of DCs which is in line with reports of SIgA getting readily internalized by DCs without any signs of maturation as evidenced by cytokine secretion or increased CD86 expression (Heystek et al., 2002). This indicates that SIgA does not interact with DCs via CD89/FcαRI but with other receptors, such as DC-SIGN. This receptor was found to specifically act on mannose-and fucose-containing carbohydrates (Geurtsen et al., 2010), which are decreased on the antibodies produced in the *N. benthamiana* ΔXT/FT plants (Göritzer et al., 2017).

When assessing the proteolytic stability of COVA2-15 variants in both saliva and BAL fluid, we observed different results between the two matrices. Several factors might contribute to this. First, the saliva sample was obtained from a human donor, whereas the BAL fluid stems from a murine source. It was reported that the composition of BAL fluid strongly varies between humans and mice (Gharib et al., 2010), with each species containing distinct proteins. Second, apart from these interspecies differences, the varying protein composition of the two fluids can cause differences in antibody stability. BAL fluid sampling targets the periphery of the lungs, specifically small-to medium-sized airways, and alveoli, whereas saliva is collected by simple expectoration from the throat. While comparative proteome analysis of these fluids displayed high similarities for immunoglobulins and albumin, which are abundant proteins in all mucosal secretions, there are many unique proteins from salivary and lower airway mucous glands (Nicholas et al., 2006). Taken together, these compositional differences can explain the varying degradation processes observed in our study.

The influence of O-glycosylation on the susceptibility of various proteins to a range of proteases is well known (Schjoldager and Clausen, 2012). It has been reported that glycosylation blocks proteolytic cleavage on recombinantly produced proteins (Herrmann et al., 1996), as indicated by increased IgG degradation by papain after removal of terminal sugars (Zheng et al., 2014) or O-glycan mediated inhibition of proteolysis of human IGFBP-6 by chymotrypsin and trypsin (Marinaro et al., 2000). Studies on O-GlcNAc-engineered peptides have revealed protective properties of this modification upon proteolysis regardless of the distant location of these residues from the actual cleavage site (Levine et al., 2019). When assaying the susceptibility of aberrantly glycosylated IgA1 typically observed in IgA nephropathy (IgAN) to several bacterial IgA proteases, it was found that IgA1 deficient of galactose, but not sialic acid was more resistant to bacterial proteolysis than normally glycosylated serum IgA1. Furthermore, different bacterial proteases showed varying enzymatic efficiencies on IgA1 immune complexes, highlighting their individual substrate specificity (Wang et al., 2016). When investigating the effect of O-glycan moieties on the IgA1 antibody variants on their susceptibility to an IgA-specific bacterial protease we observed a complete degradation of the HEK293 cell-derived sample in comparison to the plant-produced samples. GalNAc-T2 has been implicated in both increased and decreased sensitivity of diverse O-glycosylated proteins to proteolytic cleavage (King et al., 2018). In our study, the presence of mucin-type O-glycans, increased degradation by a bacterial protease in comparison to IgA1 with plant-endogenous modifications. However, more research on this topic including further proteases from other sources would be highly desirable.

In summary, we have established a novel *N. benthamiana* plant line for the recombinant production of proteins with reduced levels of HyPs and attached pentoses. We have transiently produced several variants of a highly potent anti-SARS-CoV-2 IgA1 antibody and assessed the implications of O-glycan variations in the hinge region of the antibody on its functionality. Importantly, we elucidated an influence of O-glycans on proteolytic stability and receptor- and cell-binding capacity. Given the substrate- and site-specificity of the P4H enzyme family it would be desirable to produce additional recombinant glycoproteins to determine the potentially altered or completely removed HyP residues and associated O-glycans and to evaluate their functional implications.

## Data Availability

The mass spectrometry proteomics data have been deposited to the ProteomeXchange Consortium via the PRIDE [1] partner repository with the dataset identifier PXD050139.
