# Supplementary material for: Implications of O-glycan modifications in the hinge region of a plant-produced SARS-CoV-2-IgA antibody on functionality
Source: Front Bioeng Biotechnol. 2024 Mar 6;12:1329018. doi: 10.3389/fbioe.2024.1329018 (PMC10953500; doi:10.3389/fbioe.2024.1329018)
Supplement: Supplementary file 1 [file DataSheet1.PDF]

frontiers

Supplementary Material

Implications of O-glycan modifications in the hinge region of a plant-produced SARS-CoV-2-IgA antibody on functionality

Pia Uetz, Kathrin Göritzer, Emil Vergara, Stanislav Melnik, Clemens Grünwald-Gruber, Rudolf Figl, Ala-Eddine Deghmane, Elisabetta Groppelli, Rajko Reljic, Julian K-C Ma, Eva Stöger and Richard Strasser*

*** Correspondence:** Richard Strasser: richard.strasser@boku.ac.at

1 Supplementary Figures and Tables

1.1 Supplementary Figures

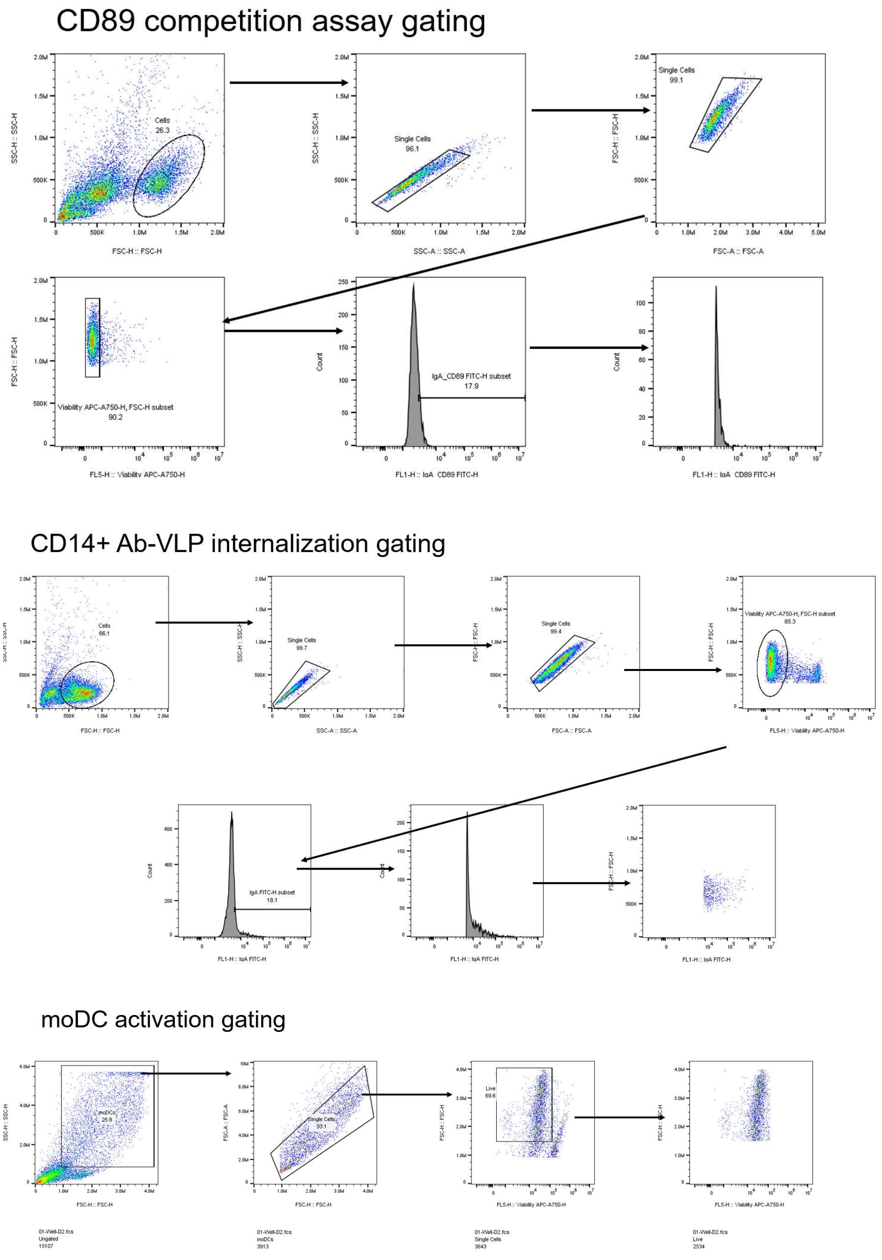

Supplementary Figure 1. Gating strategies employed for flow cytometry data analysis. Files were analyzed with FlowJoTM v10.8.1 (TreeStar, UK).

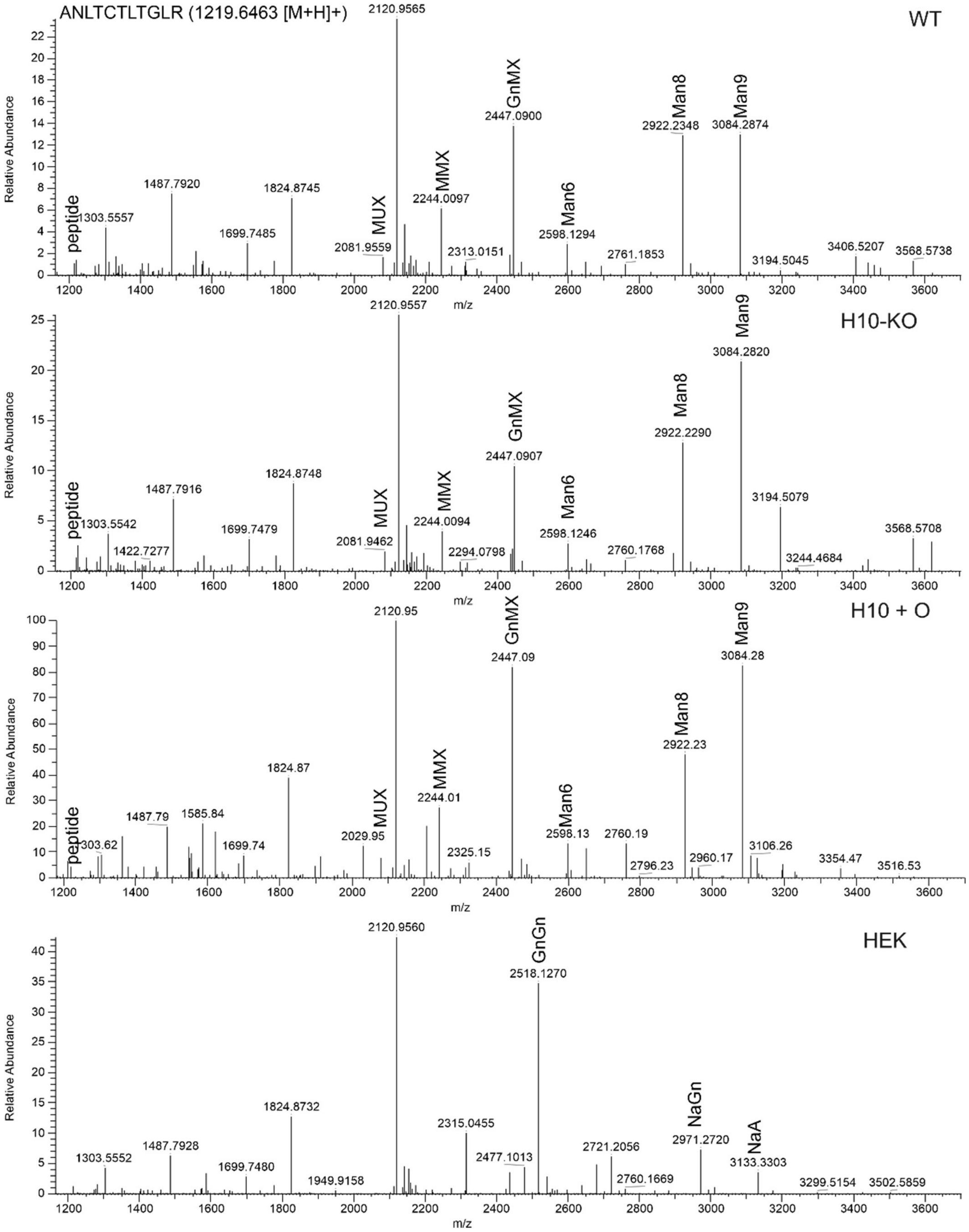

Supplementary Figure 2. LC-ESI-MS spectra of the glycopeptide carrying the CH2 domain N-glycosylation site from recombinantly produced COVA2-15 IgA1. The major peaks corresponding to the glycosylated peptide are indicated. N-glycans are abbreviated according to the ProGlycAn system (www.proglycan.com).

1.2 Supplementary Tables

Supplementary Table 1. Annotations of *NbP4H10* genes used in this study. Databases used were from the Sequencing Consortium (NbSC) (https://www.nbenth.com/), the Queensland University of Technology (QUT) (https://benthgenome.qut.edu.au/), and the Sol Genomics Network (https://solgenomics.net/). *NbP4H10_2* is represented as two separate genes within the Queensland University of Technology database.

Study-ID	Apollo LAB360	SolGenomics	QUT N.benth.
NbP4H10-1	NbL09g16660.1	Niben101Scf00083g04032.1	Nbv5.1tr6330533
NbP4H10-2	NbL06g09410.1	Niben101Scf09782g00002.1	Nbv6.1trP31841/Nbv6.1trP17689
NbP4H10-3	NbL12g07990.1	Niben101Scf08834g00008.1	Nbv6.1trP13474

Supplementary Table 2. Primers used in this study. Sequences and purpose specified.

Primer name	Primer sequence 5' → 3'	Purpose
35S_F	CTATCCTTCGCAAGACCCTTC	SpCas9 detection in transgenic plants
DM1Cas9-304_R	CGAGCCTGTGGAAGAATGAATC	
SapI-UGR_F	TGCTCTTCCATGTCGAGCTGGCAGACATACT	cloning of sgRNAs from donor vector attaching restriction sites
NbP4H10-G1_MGR	ACGTCTCTGAAGAATCAAAACCTGCCTATACGGCAGTG	
NbP4H10-G1_MGF	ACGTCTCACTTCTCGCTTTGTTTCAGAGCTATGCTGG	
NbP4H10-G2_MGR	ACGTCTCTGAAAGGAATCGACTGCCTATACGGCAGTG	
NbP4H10-G2_MGF	ACGTCTCATTTCAGTTCTAAGTTTCAGAGCTATGCTGG	
NbP4H10-G3_MGR	ACGTCTCACCTTTTCCACCACTGCCTATACGGCAGTG	
NbP4H10-G3_MGF	ACGTCTCGAAGGAGATCAGTGTTTCAGAGCTATGCTGG	
NbP4H10-G4_MGR	ACGTCTCAACTTCAGCCCACTGCCTATACGGCAGTG	
NbP4H10-G4_MGF	ACGTCTCCAAGTGATTTCATGTTTCAGAGCTATGCTGG	
NbP4H10-G5_MGF	ACGTCTCGTCTGATTAATCTGTTTCAGAGCTATGCTGG	
NbP4H10-G5_MGR	ACGTCTCACAGATATTCACACTGCCTATACGGCAGTG	
NbP4H10-G6_MGR	ACGTCTCACGAACCCTGCCCTGCCTATACGGCAGTG	
NbP4H10-G6_MGF	ACGTCTCATTCGAACAAGTTCGTTTCAGAGCTATGCTGG	
NbP4H10-G7_MGR	ACGTCTCTGATAAAGTAGTCCTGCCTATACGGCAGTG	
NbP4H10-G7_MGF	ACGTCTCCTATCTTGTCCCCGTTTCAGAGCTATGCTGG	
NbP4H10-G8_MGR	ACGTCTCAGCAGATTTTATACTGCCTATACGGCAGTG	
NbP4H10-G8_MGF	TCGTCTCACTGCATACCTACGTTTCAGAGCTATGCTGG	
SapI-UGR_R	TGCTCTTCATGCGATCCACTTGCATAGCGAGTCAG	
UV3_R	CGCAGCCGAACGACCGAGC	sequencing of sgRNAs in golden gate vector
BlaP_R	ACTCTTCCTTTTTCAATATTATTGAAGCAT	
NbP4H10-E1-2_F	GTCCAAAGTGTTTTCCATATATTGAAAC	amplification and sequencing of sgRNA target region *NbP4H0_1*
NbP4H10-E1_R	TCATTTCTTCTTTACTCTGCTGC	
NbP4H10-E2_R	GCAGAAGCAACATCACTTGA	
NbP4H10-E3_F	TGGCCATTAAAAGCATGCTATTGAGG	
NbP4H10-E3-4_F	ACATTTGCTGGTATCTAAAGAAGTCGTG	
NbP4H10-E4_F	TCTAGCACTGGATCTATTGAGCAATGG	
NbP4H10-E3_R	GAGGCATCATGGAACATTCACAGAC	
NbP4H10-E4_R	GCATAAACCTCACTGATGGAAAGTCAC	
NbP4H10_2-E1-2_F	AGCAAATTCAAAGAAGGCCACCA	amplification and sequencing of sgRNA target region *NbP4H10_2*
NbP4H10_2-E1-2_R	AGTCATATGAGCACCACTACCTGG	
NbP4H10_2-E3-4_F	TGCTGGTACCTAAGAAGTCTTTAGTAGTG	
NbP4H10_2-E4_F	CAGGATCTGTTGAGCGATGC	
NbP4H10_2-E3_R	GCCTATCTTAGATCGAGGCATCAC	
NbP4H10_2-E4_R	TCAACATCTGATCTGTGGTGCAG	

Supplementary Table 3. Guide RNAs used in this study. Target sequences within *NbP4H10* homologs were detected by CCTop software (https://cctop.cos.uni-heidelberg.de:8043/).

Guide	Sequence (5'-3')	5'PAM
G1	GTTTTGATTCTTCTCGCTTTTGG	TGG
G2	TCGATTCCTTTCAGTTCTAAAGG	AGG
G3	TGGTGGAAAAGGAGATCAGTGGG	GGG
G4	TGGGCTGAAGTGATTTCATGGG	GGG
G5	TGTGAATATCTGATTAATCTTGG	TGG
G6	GGCAGGGTTCGAACAAGTTCTGG	TGG
G7	GACTACTTTATCTTGTCCCCTGG	TGG
G8	TATAAAAATCTGCATACCTACTGG	TGG

Supplementary Table 4. Mutations induced by gRNAs. Established in a transient assay via the decomposition of sequencing chromatograms using TIDE (http://shinyapps.datacurators.nl/tide/).

Guide	total eff. (%)	major INDEL	K.O. score	R^2
NbP4H10-G1	8.1	+1 (T)	8.1	0.98
NbP4H10_2-G1	12.3	+1 (T)	12.3	0.95
NbP4H10-G2	15.8	+1 (T)	15.8	0.96
NbP4H10_2-G2	20.5	+1 (G)	20.5	0.92
NbP4H10-G3	39.4	+1 (G)	39.4	0.93
NbP4H10_2-G3	48.1	-1	42.3	0.95
NbP4H10-G4	31.6	+1 (A)	24.5	0.91
NbP4H10_2-G4	48.4	+1 (G)	41.5	0.97
NbP4H10-G5	30.9	-1	25.6	0.97
NbP4H10_2-G5	35.7	-1	29.8	0.98
NbP4H10-G6	25.1	+1 (T)	21.8	0.99
NbP4H10_2-G6	19.2	+1 (T)	16.0	0.99
NbP4H10-G7	39.0	+1 (G)	34.9	0.99
NbP4H10_2-G7	38.1	+1 (G)	35.8	0.97
NbP4H10-G8	8.4	+1 (G)	8.4	0.96
NbP4H10_2-G8	11.4	+1 (G)	11.4	0.97

Supplementary Table 5. Genome editing events detected in primary *N. benthamiana* stable transformants. Editing efficiencies and major INDELs were established using TIDE (http://shinyapps.datacurators.nl/tide/).

T0 Plant target site	total eff. (%)	INDELs	T0 Plant target site	total eff. (%)	INDELs
pB109-1A_G3	84.6	+1,+2	pB109-9_G7	44.7	-1
pB109-1A_G3B	94.1	+1,-1	pb109-9_G7B	45.9	+1
pB109-1A_G7	99.2	+1,-1	pB109-10_G3	86.9	-2
pB109-1A_G7B	71.7	+1	pB109-10_G3B	96.4	+1
pB109-1B_G3	84.2	+1,+4	pB108-10_G7	98.5	+1
pB109-1B_G3B	82.5	+1,-1	pB108-10_G7B	98.8	+1, -1
pB109-1B-G7	49.7	+1	pB109-13_G3	19.6	-2
pB109-1B_G7B	51.9	-1	pB109-13_G3B	32.9	+1
pB109-1C_G3	89.0	+1,+2,..	pB109-13_G7	33.8	+1
pB109-1C_G3B	94.8	+1,-1,..	pB109-13_G7B	32.9	+1, -1
pB109-1C_G7	50.7	+1	pB109-14_G3	80.8	-5, +1
pB109-1C_G7B	50.9	+1, -4	pB109-14_G3	95.2	+1
pB109-2_G3	18.9	-1, -3, -5	pB109-14_G7	97.6	+1
pB109-2_G3B	0	n/a	pB109-14_G7B	0	n/a
pB109-2_G7	2.6	+1,-1	pB109-15_G3	87.0	+1,-1
pB109-2_G7B	0	n/a	pB109-15_G3B	92.8	+1,-1
pB109-5_G3	7.2	-1	pB109-15_G7	47.4	-1
pB109-5_G3B	0	n/a	pB109-15_G7B	47.9	+1
pB109-5_G7	0	n/a	pB109-16_G3	79.4	+1
pB109-5_G7B	0	n/a	pB109-16_G3B	92.0	+1,-1
pB109-6_G3	78.7	-1, -8	pB109-16_G7	60.8	+1, -1
pB109-6_G3B	90.6	-1, -4	pB109-16_G7B	64.6	+1,-1
pB109-6_G7	98.5	1	pB109-17_G3	85.7	+1
pB109-6_G7B	96.5	+1, -5	pB109-17_G3B	93.1	+1, -7
pB109-9_G3	79.8	+1	pB109-17_G7	0	n/a
pB109-9_G3B	90.6	-7	pB109-17_G7B	0	n/a

Supplementary Table 6. Quantification of hinge-region O-glycans of recombinantly produced *N. benthamiana* WT and H10-KO COVA2-15 IgA1. Relative intensities (%) of glycoforms were calculated by comparing the peak intensities of the deconvoluted spectra. Three individual biological replicates were measured and the mean and standard deviation (SD) calculated.

Glycoform	H10-KO	SD	WT	SD
unmodified	55.10	1.49	8.15	1.24
1 hyPro	9.37	2.22	15.10	1.46
1 hyPro + mod	9.42	2.14	15.10	1.46
2 hyPro	2.84	0.59	16.33	2.97
2 hyPro + mod	3.72	0.35	16.46	2.97
3 hyPro	1.05	0.36	13.71	1.35
3 hyPro + mod	7.92	2.36	16.55	1.22
4 hyPro	0.46	0.09	5.99	0.84
4 hyPro + mod	18.06	1.65	18.45	0.43
5 hyPro	0.11	0.11	1.77	0.99
5 hyPro + mod	5.11	1.28	19.02	2.02
6 hyPro	0.04	0.06	0.44	0.31
6 hyPro + mod	0.41	0.24	6.16	1.36

Supplementary Table 7. Quantification of hinge-region O-glycans detected on recombinantly produced COVA2-15 IgA1 derived from H10-KO plants co-infiltrated with the human core 1-O-glycosylation pathway (H10+O) and HEK293-6E (HEK) cells. Three individual biological replicates were analyzed and relative quantities (%) were calculated from the peak intensities of the deconvoluted spectra. Hex indicates hexoses, HexNAc indicates N-acetylhexosamine, and NeuAc indicates N-acetylneuraminic acid.

Glycoform	H10+O	HEK
unmodified	8.73	0.43
Hex 1	5.10	
HexNAc	3.56	0.69
Hex 2	0.32	
Hex HexNAc	7.68	
Hex2 HexNAc1	2.61	
Hex2 HexNAc2	7.15	
HexNAc3 Hex	1.47	
HexNAc4	0.44	36.91
Hex3 HexNAc2	3.49	
HexNAc4 Hex	0.76	8.92
HexNAc5	0.37	23.06
Hex3 HexNAc3	17.13	
Hex4 HexNAc3	8.10	
Hex5 HexNAc3	5.43	
Hex4 HexNAc4	16.52	
Hex5 HexNAc4	3.88	
Hex6 HexNAc4	3.47	
Hex5 HexNAc5	3.50	
Hex6 HexNAc5	0.19	
Hex6 HexNAc6	0.10	
HexNAc2		1.18
HexNAc3		4.80
HexNAc5 Hex		9.37
HexNAc6		5.15
HexNAc4 Hex NeuAc		3.13
HexNAc6 Hex		3.20
HexNAc5 Hex NeuAc		2.45
HexNAc6 Hex NeuAc		0.71

Supplementary Table 8. Neutralization capacity of COVA2-15 IgA1 variants towards SARS-CoV-2 (England/2/2020). A plaque reduction assay in Vero E6 cells was performed and antibodies used at serial concentrations starting at 0.1 µg/mL.

IC50	
**Sample**	ng/mL
WT	0.713
H10-KO	0.485
H10+O	0.462
HEK	0.52
IgA2	0.833
IgG	1.253

Supplementary Table 9. Half-lives of COVA2-15 IgA1 variants in human saliva. Antibodies were incubated with saliva at 37°C and aliquots taken after the specified time. After performing ELISA to quantify the remaining amount of IgA1 in the aliquots a one-phase decay non-linear regression model was used to calculate the half-lives. As the values observed for the IgG variant did not plateau in the observed time frame, the calculation could not be performed (n.d., not defined).

Sample	WT	H10-KO	H10+O	HEK	IgA2	IgG
Half-life (h)	2.81	33.55	26.56	22.14	1.642	n.d.
R^2	0.95	0.98	0.99	0.99	0.92	0.70

Supplementary Table 10. Melting temperatures (V50°C) of COVA2-15 IgA1 variants and IgA2 calculated from Thermal Shift Assay (TSA). Two different buffer systems were used containing high and low salt concentrations and each sample measured in triplicate.

	PBS	Low Salt
**Sample**	V50 (°C)	
WT	72.47	71.30
H10-KO	72.61	72.10
H10 + O	72.34	72.10
HEK	72.85	71.90
IgA2	69.33	68.1
